# Dissemination of the *bla*_NDM-5_ Gene via IncX3-Type Plasmid among *Enterobacteriaceae* in Children

**DOI:** 10.1128/mSphere.00699-19

**Published:** 2020-01-08

**Authors:** Dongxing Tian, Bingjie Wang, Hong Zhang, Fen Pan, Chun Wang, Yingying Shi, Yan Sun

**Affiliations:** a Department of Clinical Laboratory, Shanghai Children’s Hospital, Shanghai Jiaotong University, Shanghai, China; Escola Paulista de Medicina/Universidade Federal de São Paulo

**Keywords:** NDM-5, *Enterobacteriaceae*, ST48, IncX3-type plasmid, carbapenemase, children, *Enterobacterales*

## Abstract

The continuous emergence of novel New Delhi metallo-β-lactamase-5 (NDM-5)-producing *Enterobacteriaceae* isolates is receiving more and more public attention. Twenty-two NDM-5-producing strains were identified from 146 carbapenemase-producing *Enterobacteriaceae* (CRE) strains isolated from pediatric patients between January and March 2017, indicating that the *bla*_NDM-5_ gene has spread to children. All 22 isolates, including 16 Klebsiella pneumoniae strains, four Klebsiella aerogenes strains, and two Escherichia coli strains, showed significantly high resistance to β-lactam antibiotics (except aztreonam) but remained susceptible to tigecycline and colistin. K. pneumoniae and *K. aerogenes* strains were respectively defined as homologous clonal isolates by pulsed-field gel electrophoresis (PFGE). Multilocus sequence typing (MLST) results confirmed the genetic relatedness with all K. pneumoniae strains belonging to sequence type (ST) 48. Two E. coli isolates (ST617 and ST1236) were considered genetically unrelated. Twenty-two *bla*_NDM-5_ plasmids were positive for the IncX3 amplicon and showed almost identical profiles after digestion with HindIII and EcoRI. Four representative strains (K. pneumoniae K725, *K. aerogenes* CR33, E. coli Z214, and E. coli Z244) were selected for further study. Plasmids harboring *bla*_NDM-5_ showed strong stability in both clinical isolates and transconjugants, without apparent plasmid loss after 100 serial generations. S1-PFGE followed by Southern blot analysis demonstrated that the *bla*_NDM-5_ gene was located on an ∼46-kb plasmid. Plasmid sequences of pNDM-K725, pNDM-CR33, and pNDM-Z214 were almost identical but were slightly different from that of pNDM-Z244. Compared with pNDM-Z244, ΔIS*Aba125* and partial copies of IS*3000* were missing. The genetic backgrounds of the *bla*_NDM-5_ gene in four strains were slightly different from that of the typical pNDM_MGR194. This study comprehensively characterized the horizontal gene transfer of the *bla*_NDM-5_ gene among different *Enterobacteriaceae* isolates in pediatric patients, and the IncX3-type plasmid was responsible for the spread.

**IMPORTANCE** The emergence of CRE strains resistant to multiple antibiotics is considered a substantial threat to human health. Therefore, all the efforts to provide a detailed molecular transmission mechanism of specific drug resistance can contribute positively to prevent the further spread of multidrug-resistant bacteria. Although the new superbug harboring *bla*_NDM-5_ has been reported in many countries, it was mostly identified among E. coli strains, and the gene transfer mechanism has not been fully recognized and studied. In this work, we identified 22 *bla*_NDM-5_-positive strains in different species of *Enterobacteriaceae*, including 16 Klebsiella pneumoniae strains, four Klebsiella aerogenes strains, and two Escherichia coli strains, which indicated the horizontal gene transfer of *bla*_NDM-5_ among *Enterobacteriaceae* strains in pediatric patients. Moreover, *bla*_NDM-5_ was located on a 46-kb IncX3 plasmid, which is possibly responsible for this widespread horizontal gene transfer. The different genetic contexts of the *bla*_NDM-5_ gene indicated some minor evolutions of the plasmid, based on the complete sequences of the *bla*_NDM-5_ plasmids. These findings are of great significance to understand the transmission mechanism of drug resistance genes, develop anti-infection treatment, and take effective infection control measures.

## Abstract (precis)

The emergence of CRE strains resistant to multiple antibiotics is considered a substantial threat to human health. Therefore, all the efforts to provide a detailed molecular transmission mechanism of specific drug resistance can contribute positively to prevent the further spread of multidrug-resistant bacteria. Although the new superbug harboring *bla*_NDM-5_ has been reported in many countries, it was mostly identified among E. coli strains, and the gene transfer mechanism has not been fully recognized and studied. In this work, we identified 22 *bla*_NDM-5_-positive strains in different species of *Enterobacteriaceae*, including 16 Klebsiella pneumoniae strains, four Klebsiella aerogenes strains, and two Escherichia coli strains, which indicated the horizontal gene transfer of *bla*_NDM-5_ among *Enterobacteriaceae* strains in pediatric patients. Moreover, *bla*_NDM-5_ was located on a 46-kb IncX3 plasmid, which is possibly responsible for this widespread horizontal gene transfer. The different genetic contexts of the *bla*_NDM-5_ gene indicated some minor evolutions of the plasmid, based on the complete sequences of the *bla*_NDM-5_ plasmids. These findings are of great significance to understand the transmission mechanism of drug resistance genes, develop anti-infection treatment, and take effective infection control measures.

## INTRODUCTION

Carbapenemase-producing *Enterobacteriaceae* (CRE) have become a serious challenge to clinical therapy owing to the rapid worldwide dissemination of multidrug resistance (MDR) (1). New Delhi metallo-β-lactamase (NDM) is the main carbapenemase detected in children (2), which is capable of hydrolyzing almost all β-lactams and has the potential to cause a global health crisis. Since the first report of NDM-1, 21 variants of NDM enzymes (NDM-1 to NDM-21) have been identified worldwide (3).

New Delhi metallo-β-lactamase-5 (NDM-5) was first identified in a multidrug-resistant Escherichia coli ST648 isolate in the United Kingdom in 2011 (4). Since then, NDM-5 has been reported all over the world, including in Egypt (5), South Korea (6), China (7), the United States (8), Italy (9), and Spain (10). However, NDM-5 has mainly been identified in E. coli and a few other *Enterobacteriaceae* isolates (11). The NDM-5 enzyme differs from NDM-1 by only two amino acid substitutions (Val88Leu and Met154Leu) and shows increased resistance to carbapenems and broad-spectrum cephalosporins (4). It is a concern that *bla*_NDM-5_ was detected in not only clinical specimens but also animals (12, 13) and environmental samples (14), indicating its potential to spread further in the community. The *bla*_NDM-5_ gene was reported to be carried in different incompatibility typing plasmids to transfer genes such as IncFII, IncX3, IncN, and IncF (15). A fusion plasmid (IncX3 and IncFIB) bearing *bla*_NDM-5_ in E. coli was also identified (16, 17). These plasmids can facilitate the spread of *bla*_NDM-5_ in *Enterobacteriaceae* through horizontal gene transfer.

In this study, we screened NDM-5-producing *Enterobacteriaceae* strains in pediatric patients to elucidate the dissemination mechanism and provided the complete sequence of IncX3 plasmids to confirm the horizontal gene transfer of *bla*_NDM-5_ among *Enterobacteriaceae*. In addition, to the best of our knowledge, this is the first time that clonal dissemination of NDM-5-producing ST48 Klebsiella pneumoniae and Klebsiella aerogenes has been reported in children.

## RESULTS

### Bacterial strains and antimicrobial susceptibility testing.

Among 146 CRE isolates, 22 *bla*_NDM-5_-positive *Enterobacteriaceae* isolates were identified, including 16 K. pneumoniae, four *K. aerogenes*, and two E. coli isolates. The distributions of other carbapenemase genes are shown in Table S1 in the supplemental material and not discussed in this study. The *bla*_NDM-5_-positive isolates were all recovered from patients on the neonatal intensive care unit (NICU) or pediatric intensive care unit (PICU) wards and were mainly collected from blood and sputum samples. All isolates showed high resistance to β-lactam antibiotics and inhibitors (except aztreonam), including imipenem, meropenem, ertapenem, cefotaxime, cefepime, ceftazidime, cefmetazole, piperacillin-tazobactam, and ceftazidime-avibactam. Most strains showed resistance to sulfamethoxazole-trimethoprim but always remained susceptible to tigecycline and colistin; most were susceptible to amikacin, gentamicin, ciprofloxacin, levofloxacin, and aztreonam (Table 1).

**TABLE 1:** Antimicrobial susceptibility of NDM-5-producing *Enterobacteriaceae* isolates

Isolate	Species	MIC (μg/ml) of drug^*a*^:																	
		ETP	IPM	MEM	AMK	GEN	SXT	LVX	CIP	CTX	FEP	CAZ	CMZ	TZP	CSL	CZA	ATM	TGC	COL
K24	K. pneumoniae	256	128	256	1	0.5	>256/4,864	2	2	>32	>32	>32	>64	>256	>128	>128	>128	≤0.125	0.25
K32	K. pneumoniae	256	128	256	1	0.25	>256/4,864	0.5	1	>32	>32	>32	>64	>256	>128	>128	>128	≤0.125	0.25
K158	K. pneumoniae	128	64	256	1	0.25	>256/4,864	0.5	1	>32	>32	>32	>64	>256	>128	>128	>128	≤0.125	0.25
K27	K. pneumoniae	256	256	256	2	0.5	>256/4,864	0.5	1	>32	>32	>32	>64	>256	>128	>128	>128	≤0.125	0.25
K176	K. pneumoniae	256	256	256	1	0.5	>256/4,864	0.5	1	>32	>32	>32	>64	>256	>128	>128	>128	0.25	0.5
K178	K. pneumoniae	256	128	256	2	0.25	>256/4,864	1	1	>32	>32	>32	>64	>256	>128	>128	>128	≤0.125	0.25
K182	K. pneumoniae	256	256	256	2	0.25	>256/4,864	0.5	0.5	>32	>32	>32	>64	>256	>128	>128	>128	≤0.125	1
K183	K. pneumoniae	256	128	256	0.5	≤0.125	>256/4,864	1	1	>32	>32	>32	>64	>256	>128	128	>128	0.25	0.25
K184	K. pneumoniae	256	128	256	2	0.5	>256/4,864	1	1	>32	>32	>32	>64	>256	>128	64	128	≤0.125	0.25
K161	K. pneumoniae	256	256	256	>512	>256	>256/4,864	16	32	>32	>32	>32	>64	>256	>128	>128	>128	≤0.125	0.25
K185	K. pneumoniae	256	256	256	2	0.5	>256/4,864	≤0.125	≤0.125	>32	>32	>32	>64	>256	>128	>128	>128	≤0.125	2
K187	K. pneumoniae	256	128	256	2	0.5	>256/4,864	1	1	>32	>32	>32	>64	>256	>128	128	>128	≤0.125	0.5
K45	K. pneumoniae	256	128	256	1	0.25	>256/4,864	0.5	1	>32	>32	>32	>64	>256	>128	>128	128	≤0.125	0.25
K96	K. pneumoniae	>256	128	256	2	0.5	>256/4,864	0.5	0.5	>32	>32	>32	>64	>256	>128	>128	>128	≤0.125	0.25
K702	K. pneumoniae	256	256	256	1	0.25	>256/4,864	1	1	>32	>32	>32	>64	>256	>128	>128	>128	≤0.125	0.25
K725	K. pneumoniae	128	64	256	1	0.25	>256/4,864	1	1	>32	>32	>32	>64	>256	>128	>128	>128	≤0.125	0.25
Z214	E. coli	16	16	32	2	0.5	>256/4,864	0.5	0.5	>32	>32	>32	>64	>256	>128	32	≤1	≤0.125	≤0.125
Z244	E. coli	128	32	128	4	32	≤0.125/2.4	16	64	>32	>32	>32	64	>256	>128	16	>128	≤0.125	≤0.125
CR33	*K. aerogenes*	64	64	128	2	0.5	0.5/9.5	1	1	>32	>32	>32	>64	>256	>128	>128	≤1	≤0.125	≤0.125
CR94	*K. aerogenes*	64	64	128	2	0.5	0.5/9.5	1	1	>32	>32	>32	>64	>256	>128	>128	32	≤0.125	0.25
CR39	*K. aerogenes*	64	64	128	2	0.5	0.25/4.7	1	0.5	>32	>32	>32	>64	>256	>128	>128	32	≤0.125	0.25
CR50	*K. aerogenes*	64	32	128	2	0.25	≤0.125/2.4	1	1	>32	>32	>32	>64	>256	>128	>128	32	≤0.125	0.25

a Abbreviations: ETP, ertapenem; IPM, imipenem; MEM, meropenem; AMK, amikacin; GEN, gentamicin; SXT, sulfamethoxazole-trimethoprim; LVX, levofloxacin; CIP, ciprofloxacin; CTX, cefotaxime; FEP, cefepime; CAZ, ceftazidime; CMZ, cefmetazole; TZP, piperacillin-tazobactam; CSL, cefoperazone-sulbactam; CZA, ceftazidime-avibactam; ATM, aztreonam; TGC, tigecycline; COL, colistin.

10.1128/mSphere.00699-19.1 TABLE S1 Distribution of carbapenemase genes in *Enterobacteriaceae* strains. Download Table S1, DOCX file, 0.02 MB. Copyright © 2020 Tian et al. 2020 Tian et al. This content is distributed under the terms of the Creative Commons Attribution 4.0 International license.

### Genetic relatedness.

**FIG 1:**
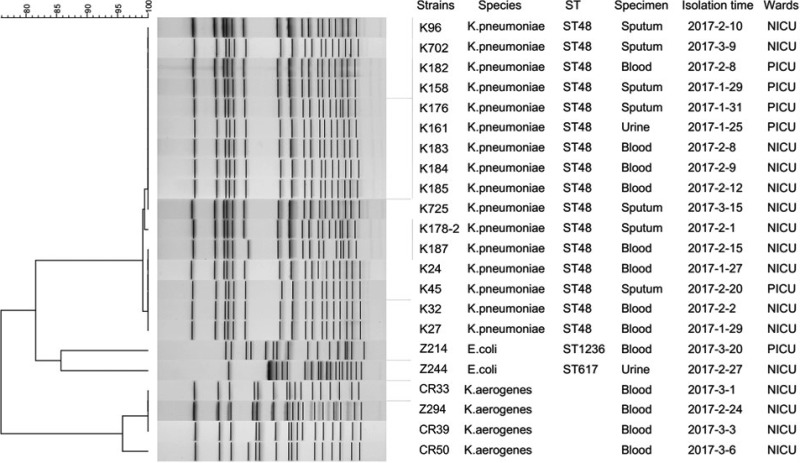
PFGE profiles and MLST results for NDM-5-producing *Enterobacteriaceae* isolates.

Multilocus sequence typing (MLST) and pulsed-field gel electrophoresis (PFGE) experiments were performed to analyze the clonal relatedness of NDM-5-producing *Enterobacteriaceae* isolates because *bla*_NDM-5_-positive isolates are not common in children. According to the MLST results, 16 K. pneumoniae isolates belonged to the same type, ST48, and two E. coli isolates belonged to ST617 and ST1236, respectively (Fig. 1). Sequence typing of *K. aerogenes* isolates was not performed because it has not been well established for this organism. In accordance with the MLST results, PFGE patterns confirmed the close genetic relatedness of 16 K. pneumoniae isolates, and four *K. aerogenes* isolates also had similar PFGE profiles (Fig. 1). Two E. coli isolates had different PFGE patterns (Fig. 1).

### Characterization of the *bla*_NDM-5_ gene.

**FIG 2:**
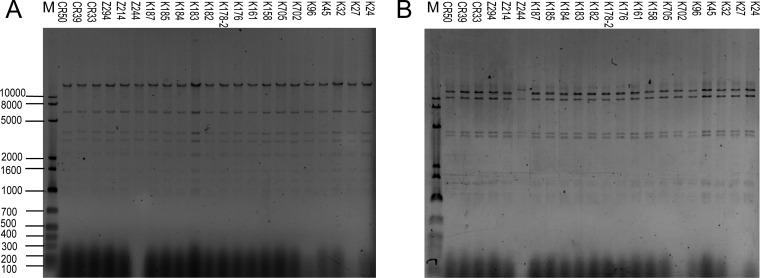
RFLP analysis of *bla*_NDM-5_-positive plasmids. (A) *bla*_NDM-5_-positive plasmids digested with EcoRI. (B) *bla*_NDM-5_-positive plasmids digested with HindIII. Lane M, 1 kb plus DNA ladder marker.

The plasmids carrying the *bla*_NDM-5_ gene of 22 *Enterobacteriaceae* isolates were successfully transferred into recipient E. coli J53 with a conjugation rate of ∼10^−3^ per receipt strain. Compared to E. coli J53, the transconjugants exhibited significantly increased resistance to carbapenems (Table S2). Twenty-two *bla*_NDM-5_ plasmids were positive for the IncX3 amplicon and negative for other plasmid types. Plasmids digested with EcoRI showed the same profiles, but the plasmid in Z244 isolates showed some differences when digested with HindIII (Fig. 2). Therefore, according to the restriction fragment length polymorphism (RFLP) of plasmids and genetic relatedness, K. pneumoniae K725, *K. aerogenes* CR33, E. coli Z214, and E. coli Z244 were selected for further study.

10.1128/mSphere.00699-19.2 TABLE S2 Antimicrobial susceptibility of *bla*_NDM-5_-positive transconjugants. Download Table S2, DOCX file, 0.02 MB. Copyright © 2020 Tian et al. 2020 Tian et al. This content is distributed under the terms of the Creative Commons Attribution 4.0 International license.

**FIG 3:**
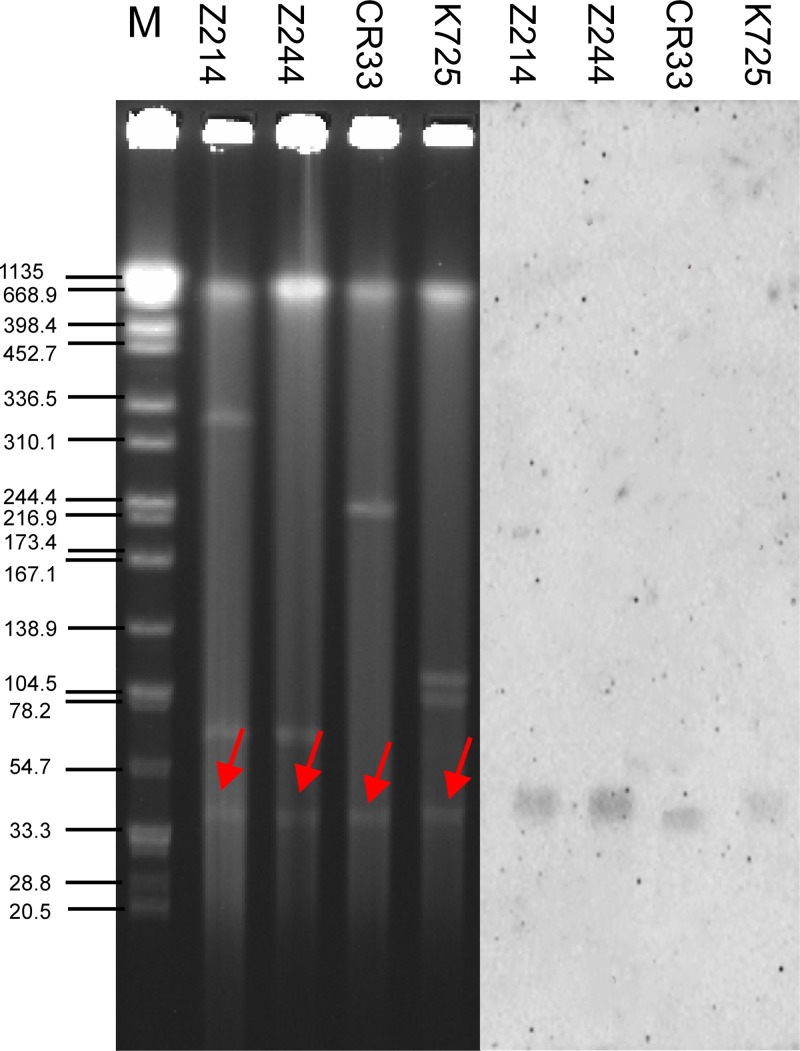
S1-digested plasmid DNA and Southern blot hybridization. Bands indicated with arrows showed positive signals in Southern blot hybridization with the *bla*_NDM-5_ probe. Lane M, *Salmonella* serotype Braenderup strain H9812 molecular marker.

Plasmids harboring *bla*_NDM-5_ showed strong stability in both clinical isolates and transconjugants, without apparent plasmid loss after 100 serial generations (data not shown). The results of S1-PFGE followed by Southern blot analysis demonstrated that K. pneumoniae K725 contains three plasmids (46, 70, and 320 kb), *K. aerogenes* CR33 contains two plasmids (46 and 70 kb), E. coli Z244 contains two plasmids (46 and 230 kb), and E. coli Z214 contains three plasmids (46, 90, and 115 kb). *bla*_NDM-5_ genes are all located on plasmids of similar size (∼46 kb) (Fig. 3).

### Plasmid sequence and comparative analysis.

**FIG 4:**
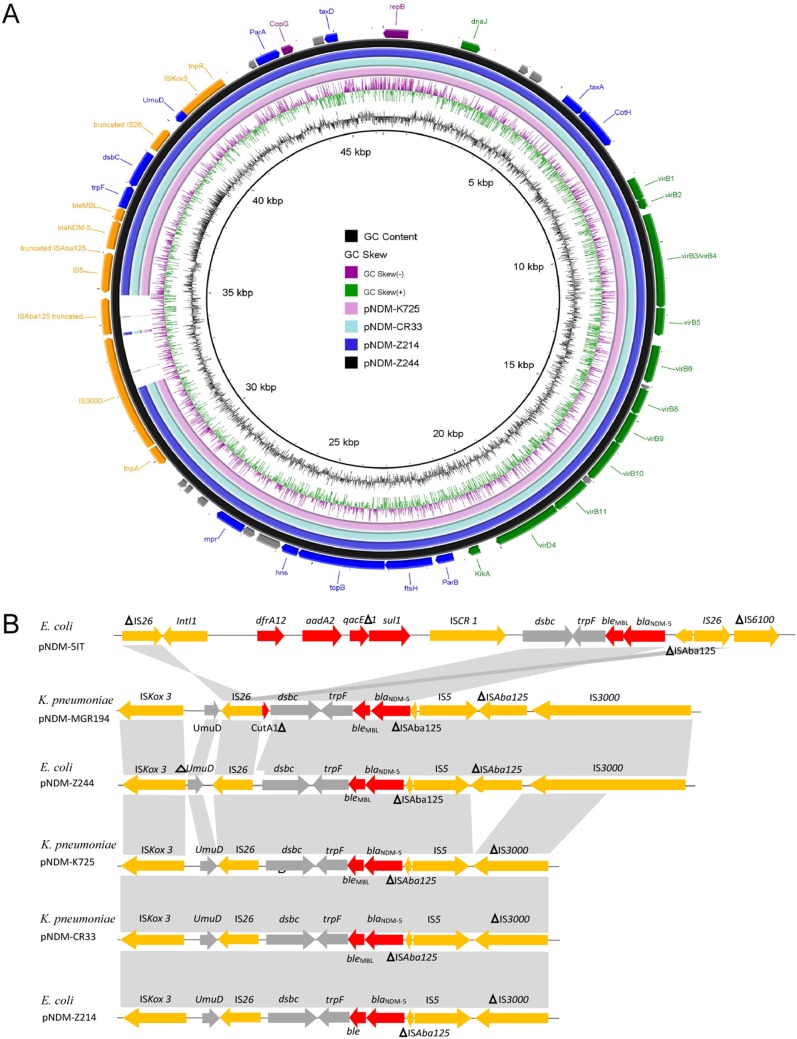
Sequence analysis of *bla*_NDM-5_-positive plasmids. (A) Comparative analysis of pNDM-K725, pNDM-CR33, pNDM-Z214, and pNDM-Z244. The circular map was created by BRIG tools. Concentric rings represent the similarity between the reference sequence (pNDM-Z244) in the outer ring and other sequences in the inner rings. Color levels indicate the results of BLAST with a matched degree in the shared regions. Genes shown in purple, blue, green, and orange are involved in replication, stability, propagation, and application, respectively. Genes encoding unknown functions or those not directly related to the above-mentioned roles are indicated in gray and are shown unlabeled. (B) Comparative analysis of the genetic contexts of *bla*_NDM-5_ in pNDM-5-IT (GenBank accession no. MG649062), pNDM-MGR194 (GenBank accession no. KF220657), pNDM-Z244, pNDM-K725, pNDM-KZ214, and pNDM-CR33. The putative open reading frames are shown as arrowheads according to the direction of transcription. Regions with similar sequences are indicated in gray between the different plasmids.

The entire plasmid sequences were obtained to characterize the *bla*_NDM-5_ plasmid better and enable comparative analyses in K. pneumoniae, *K. aerogenes*, and E. coli isolates. The plasmids of K725, CR33, Z214, and Z244 were 43,125, 43,252, 43,252, and 46,047 bp in length, respectively, all belonging to the IncX3 incompatibility plasmid type. Comparative analysis showed almost identical sequences among pNDM-K725, pNDM-CR33, and pNDM-Z214 but a slight difference from pNDM-Z244 (Fig. 4A). Compared with pNDM-Z244, IS*Aba125* truncated by IS*5* was almost missing with only 73 bp remaining, and partial copies of IS*3000* were also deleted in pNDM-K725, pNDM-CR33, and pNDM-Z214 (Fig. 4B). The above deletions resulted in the smaller size of pNDM-K725, pNDM-CR33, and pNDM-Z214. The complete sequence of pNDM-Z244 was used as a reference to draw a circular map of the plasmids in four isolates. Using the approach of Norman and colleagues (35), pNDM-Z244 was determined to carry genes involved in replication (*repB* and *copG*), stability (*taxA*, *cotH*, *parB*, *ftsH*, *topB*, *hns*, *mpr*, *trpF*, *dsbC*, *umuD*, *parA*, and *taxD*), propagation (*dnaJ*, *virB1*, *virB2*, *virB3*/*virB4*, *virB5*, *virB6*, *virB8*, *virB9*, *virB10*, *virB11*, *virD4*, and *kikA*), and adaptation (*tnpA*-IS*3000*-IS*Aba125*-IS*5*-*bla*_NDM-5_-*bla*_MBL_-IS*26*-IS*Kox3*). The plasmid harboring 67 predicted open reading frames (ORFs) contained only one resistance gene, *bla*_NDM-5_ (Fig. 4A), indicating that antibiotic resistance genes in other plasmids may be responsible for the resistance to a variety of antibiotics.

The *bla*_NDM-5_ gene was flanked in the upstream region *by* IS*3000-Δ*IS*Aba125-*IS*5-Δ*IS*Aba125* and downstream by *ble*_MBL_-*trpF*-*dsbC*-IS*26*-Δ*umuD*-IS*Kox3*, and this genetic background is the same as that of isolate pNDM_MGR194 in India (GenBank accession no. KF220657) (Fig. 4B) (18). Deletions of IS*Aba125* and IS*3100* in plasmids pNDM-K725, pNDM-CR33, and pNDM-Z214 suggest that additional gene deletions and rearrangements may occur in these plasmids. The *bla*_NDM-5_ gene within pNDM-5-IT (GenBank accession no. MG649062), which was detected in Italy, was located in a complex integron, bracketed by two IS*26* sequences containing an IS*CR1* element and a class 1 integron with the *intI1* gene truncated by one of the IS*26* copies and the *aadA2*-*dfrA12* resistance gene cassettes (Fig. 4B).

## DISCUSSION

To date, NDM-5 carbapenemase has been described mostly in E. coli and rarely in K. pneumoniae and other *Enterobacteriaceae* isolates (11). Furthermore, to the best of our knowledge, a neonatal outbreak of NDM-5-producing Klebsiella quasipneumoniae in Nigeria was recently reported, but other clonal dissemination of *bla*_NDM-5_ was very rarely found in children (19). In this study, we reported the dissemination of *bla*_NDM-5_ among different species of *Enterobacteriaceae* in children, including E. coli, K. pneumoniae, and *K. aerogenes.* Although NDM-5-producing strains are not as widespread as NDM-1-producing strains, they can also accompany multiple resistance gene determinants of resistance to different antimicrobials in the same strain, which makes them a potential public health threat. Furthermore, *bla*_NDM-5_ can occasionally occur simultaneously with *bla*_OXA-181_ (20–22), but *bla*_OXA-181_ was not found in our study. The NDM-5-producing strains described in our study showed high resistance to all β-lactams and inhibitors. Furthermore, most of them remained susceptible to aminoglycosides and fluoroquinolones, a finding which is not consistent with NDM producers usually also being resistant to aminoglycosides because they frequently harbor 16S rRNA methylases, such as *armA* and *rmtB* (23–25). The rare clinical usage of these drugs in children owing to their side effects may be the reason for the susceptibility to aminoglycosides and fluoroquinolones observed in our study. Fortunately, strains resistant to tigecycline and colistin were not found.

K. pneumoniae is one of the most important pathogens threatening children’s health. The emergence of the *bla*_NDM-5_ gene in K. pneumoniae increased the difficulty of clinical treatment of this pathogen. Sixteen K. pneumoniae isolates carrying *bla*_NDM-5_ in our study belonged to the same sequence type, ST48, and had similar PFGE profiles, strongly indicating that clonal dissemination of K. pneumoniae carrying *bla*_NDM-5_ had occurred in our hospital. To our knowledge, *bla*_NDM-5_-positive K. pneumoniae isolated from clinical samples has been identified in ST2250 in China (7), ST2266 in New Zealand (26), ST147 in the United States (21), and ST231 in Singapore (22) and in untypeable isolates in India (18). This is very possibly the first report in the world of ST48 carbapenem-resistant K. pneumoniae carrying the *bla*_NDM-5_ gene.

Significantly, the *bla*_NDM-5_ gene was also found in two E. coli strains and four *K. aerogenes* strains. Four *K. aerogenes* strains with identical PFGE profiles were possibly caused by clonal dissemination, while two distantly related E. coli strains (ST617 and ST1236) may have acquired the *bla*_NDM-5_-positive plasmid by horizontal transfer. Previous studies suggested that the *bla*_NDM-5_ gene has been most frequently detected in E. coli of many sequence types, with the most common being ST167 (11), whereas E. coli carrying the *bla*_NDM-5_ gene detected in this study belonged to ST617. Interestingly, one study characterized ST167 and ST617 as sister clades with respect to ST10, with ST617 emerging as a nested clade from a single outlying ST167 genome (27). The study also indicated that lineage-specific alterations in intergenic regions were responsible for the emergence of the multidrug resistance (MDR) plasmid. Therefore, there is a need for a more thorough and detailed analysis of the genomic epidemiological investigation of bacteria carrying carbapenem resistance plasmids. Notably, most of the strains were collected from blood samples. Unlike other types of infection, bloodstream infections are always associated with high mortality. Therefore, we should be vigilant in preventing a further spread of the *bla*_NDM-5_ gene in other *Enterobacteriaceae* isolates.

The *bla*_NDM-5_ gene has previously been reported to be carried on a 46-kb self-transmissible plasmid, which belongs to the IncX3 incompatible group. The results of plasmid sequencing in our study revealed that plasmid pNDM-Z244 in E. coli was mostly identical to pNDM-MGR194 reported in India, except for several mutations (18). Plasmids pNDM-K725, pNDM-CR33, and pNDM-Z214 were mostly identical to each other but were slightly different from pNDM-Z244. We speculated that E. coli Z244 possibly acquired the *bla*_NDM-5_ gene from commonly reported plasmids like pNDM-MGR194, which can also be found in strains isolated from environmental, animal, and human clinical samples (12, 14, 28). A study revealing that NDM-5-producing E. coli ST167 was simultaneously detected in a companion dog and his owners in a family in Finland (29) indicated that human-to-canine transmission is possible. Therefore, it may be logical to assume that some *Enterobacteriaceae* strains acquired the *bla*_NDM-5_-positive plasmid by horizontal transfer, and it was further clonally disseminated, which resulted in this outbreak of the *bla*_NDM-5_ gene in our hospital.

Interaction with the host or the adaptation response during horizontal transfer possibly resulted in the loss of IS*Aba125* and part of IS*3000* sequences. IS*Aba125* was always found in Acinetobacter spp. and was mainly embedded in the chromosome (30). Currently, it is widely accepted that the *bla*_NDM_ gene is transferred from Acinetobacter spp. to *Enterobacteriaceae* through I*SAba125* and IS*26* or other transposable elements (31). That the IncX3-type plasmid spreads easily in *Enterobacteriaceae* may be responsible for the dissemination of the *bla*_NDM-5_ gene. Transposable elements such as IS*Aba125* were not the main factor, nor were they essential for plasmid replication and proliferation or stability of host strains, so IS*Aba125* could be gradually deleted in the process of transfer. Previous studies have reported a partial loss of IS*Aba125* around *bla*_NDM-5_ (7, 15), but the almost complete loss is reported for the first time. More experiments are needed to confirm whether this microevolution contributes to the plasmid transfer. The IncX3-type plasmid was also frequently reported to mediate the dissemination of other NDM variants, including *bla*_NDM-1_, *bla*_NDM-4_, *bla*_NDM-7_, *bla*_NDM-13_, *bla*_NDM-17_, *bla*_NDM-19_, *bla*_NDM-20_, and *bla*_NDM-21_ (3, 15), which indicated that the *bla*_NDM_-bearing IncX3-type plasmids might have evolved from the same ancestral plasmid through a series of mutations. Easy spread of the IncX3-type plasmid could be responsible for the dissemination of multiple NDM variants in *Enterobacteriaceae* isolates.

Plasmids in this study harbored only one resistance gene, *bla*_NDM-5_, which had a similar genetic background except that part of IS*3000* and IS*Aba125* remnants were deleted in pNDM-K725, pNDM-CR33, and pNDM-Z214. According to the complete sequences of the plasmids, the genetic background of *bla*_NDM-5_ in pNDM-Z244 was similar to that in the classical plasmid pNDM-MGR194, i.e., IS*3000*-IS*5*-*Δ*IS*Aba125*-*bla*_NDM-5_-*bla*_MBL_-*trpF*-*dsbC*-IS*26*-*umuD*. In contrast, the *bla*_NDM-5_ gene in pNDM-5-IT was more complex and was found in the *dfrA12*-*aadA2*-IS*CR1*-*bla*_NDM-5_ complex integron (9).

In conclusion, we characterized the IncX3-type plasmid carrying the *bla*_NDM-5_ gene of K. pneumoniae, E. coli, and *K. aerogenes* clinical isolates. Our results may serve as evidence of horizontal gene transfer of *bla*_NDM-5_ among different *Enterobacteriaceae* isolates. To our knowledge, this is the first report of *bla*_NDM-5_-carrying isolates in different species of *Enterobacteriaceae* in pediatric patients in China.

## MATERIALS AND METHODS

### Bacterial strains.

A total of 146 carbapenem-resistant *Enterobacteriaceae* (CRE) strains were collected between January and March 2017 in a children’s hospital in Shanghai, China. They were mainly isolated from nasopharyngeal secretions, blood, pus secretions, urine, catheter, and ascites. The protocol was approved by the Ethics Committee of Shanghai Children’s Hospital, Shanghai Jiaotong University. Individual informed consent was waived because we used existing strains and did not pose any additional risks to the patients. Matrix-assisted laser desorption ionization–time of flight (MALDI-TOF) mass spectrometry (Bruker Daltonics GmBH, Bremen, Germany) was used for bacterial identification, and disc diffusion assays (for imipenem and meropenem) were used to identify carbapenem resistance. Common carbapenemase genes (*bla*_KPC_, *bla*_NDM_, *bla*_IMP_, *bla*_OXA-48_, *bla*_VIM_, *bla*_AIM_, *bla*_GIM_, and *bla*_SIM_) were amplified for all strains using primers from the previous study, and the positive products were sequenced (2). Twenty-two *bla*_NDM-5_-positive strains were finally selected for further study.

### Antimicrobial susceptibility testing.

Antimicrobial susceptibility was determined using the broth microdilution method according to the guidelines of the Clinical and Laboratory Standards Institute (CLSI) (32). The antibiotics tested were ertapenem, imipenem, meropenem, ceftazidime, cefotaxime, cefmetazole, cefepime, piperacillin-tazobactam, cefoperazone-sulbactam, ceftazidime-avibactam, amikacin, gentamicin, nitrofurantoin, sulfamethoxazole-trimethoprim, aztreonam, ciprofloxacin, levofloxacin, tigecycline, polymyxin, and colistin. The results were determined and interpreted as follows: colistin and tigecycline according to the European Committee on Antimicrobial Susceptibility Testing (EUCAST) (36) and all others according to the CLSI M100-S28 criteria (32). E. coli ATCC 25922 was used for quality control.

### Determination of genetic relatedness.

MLST was determined using the platform for K. pneumoniae MLST maintained at the Institut Pasteur, Paris, France (https://bigsdb.pasteur.fr/klebsiella/primers_used.html) and E. coli MLST maintained at the Achtman multilocus sequence typing scheme (http://mlst.warwick.ac.uk/mlst/dbs/Ecoli/documents/primersColi_html). Seven housekeeping genes of E. coli and K. pneumoniae were amplified by PCR, and the products were sequenced to analyze the ST. PFGE was further performed according to previously defined criteria (33). Briefly, the isolates were digested by XbaI endonuclease and analyzed using a CHEF-Mapper XA PFGE system (Bio-Rad, CA, USA) with a 2.16- to 54.17-s linear ramp for 19 h at 6 V/cm and 14°C. The PFGE profiles were analyzed with BioNumerics software (Applied Maths, Sint-Martens-Latem, Belgium). Salmonella enterica serotype Braenderup H9812 was used as a size marker.

### Plasmid analysis and location of *bla*_NDM-5_.

Filter-mating conjugation experiments were performed between 22 different isolates. E. coli J53 resistant to sodium azide was used as the recipient strain. Transconjugants that possessed the *bla*_NDM-5_-bearing plasmid were selected on Mueller-Hinton agar (MHA; Oxoid) plates that contained 180 μg/ml sodium azide with 1 μg/ml meropenem. Antimicrobial susceptibility testing and PCR amplification of the transconjugants were subsequently performed to confirm whether the plasmid was successfully transferred to the recipient. The PBRT 2.0 kit for PCR-based replicon typing was used for molecular typing of plasmids (Diatheva, Fano, Italy). Plasmid relationships were tested by restriction fragment length polymorphism (RFLP) using HindIII and EcoRI. Digested plasmid DNA was electrophoresed in a 0.8% agarose gel for approximately 1 h. Four strains were selected for further study. Plasmid stability was tested by liquid experiments as previously described (34). S1-PFGE and Southern blotting were further performed to determine the plasmid location of the *bla*_NDM-5_ gene. Genomic DNA digested with S1 nuclease was subjected to PFGE as described above. The DNA fragments were transferred to a positively charged nylon membrane (Millipore, USA) and then hybridized with a digoxigenin-labeled NDM-5-specific probe. S. enterica serotype Braenderup H9812 was used as the size marker.

### Plasmid sequencing and comparative analysis.

To obtain a comprehensive understanding of the plasmid carrying the *bla*_NDM-5_ gene, complete sequencing was further performed. The plasmid DNAs of transconjugants were extracted using a HiSpeed Plasmid Midi kit (Qiagen, Valencia, CA, USA) according to the manufacturer’s recommendations. The plasmids were sequenced on an Illumina MiSeq 2000 (Illumina Inc., San Diego, CA, USA) platform with 2- by 300-bp paired-end reads. The raw data quality control was performed with FastQC software (v. 0.11.8, http://www.bioinformatics.babraham.ac.uk/projects/fastqc/). The clean reads were assembled using SPAdesv3.9.0 and A5-miseq v20150522. Prediction and annotation of the open reading frames (ORFs) were carried out using the RAST (Rapid Annotation using Subsystems Technology) website server (http://rast.nmpdr.org/). BRIG was used in comparative analysis and the generation of plasmid maps.

### Data availability.

The complete sequences of the plasmids were submitted to the National Center for Biotechnology Information (NCBI) database under the accession numbers in parentheses: pNDM-K725 (MK450348), pNDM-CR33 (MK450349), pNDM-Z214 (MK450347), and pNDM-Z244 (MK450346).
