# Supplementary material for: Dissemination of the blaNDM-5 Gene via IncX3-Type Plasmid among Enterobacteriaceae in Children
Source: mSphere. 2020 Jan 8;5(1):e00699-19. doi: 10.1128/mSphere.00699-19 (PMC6952193; doi:10.1128/mSphere.00699-19)
Supplement: TABLE S1 [file mSphere.00699-19-st001.docx]

Table S1

| **Carbapenemase genes** | **Number of strains** | ***Klebsiella pneumoniae*** | ***Escherichia coli*** | ***Enterobacter cloacae*** | ***Klebsiella aerogenes*** |
| --- | --- | --- | --- | --- | --- |
| *bla*_KPC-2_ | 40 | 37 | 3 | 0 | 0 |
| *bla*_NDM-5_ | 22 | 16 | 2 | 0 | 4 |
| *bla*_NDM-1_ | 25 | 8 | 9 | 8 | 0 |
| *bla*_IMP_ | 14 | 4 | 0 | 10 | 0 |
| *bla*_OXA-232_ | 36 | 36 | 0 | 0 | 0 |
| *bla*_NDM_+*bla*_KPC-2_ | 1 | 1 | 0 | 0 | 0 |
| *bla*_KPC-2_+*bla*_IMP_ | 1 | 1 | 0 | 0 | 0 |
| undefined | 8 | 3 | 2 | 3 | 0 |
| total | 147 | 106 | 16 | 21 | 4 |
