# Supplementary material for: Dissemination of the blaNDM-5 Gene via IncX3-Type Plasmid among Enterobacteriaceae in Children
Source: mSphere. 2020 Jan 8;5(1):e00699-19. doi: 10.1128/mSphere.00699-19 (PMC6952193; doi:10.1128/mSphere.00699-19)
Supplement: TABLE S2 [file mSphere.00699-19-st002.docx]

Table S2

| Isolates | Species | MIC (μg/mL) | | | | | | | | | | | | | | | | | |
| --- | --- | --- | --- | --- | --- | --- | --- | --- | --- | --- | --- | --- | --- | --- | --- | --- | --- | --- | --- |
|  |  | ETP | IPM | MEM | AMK | GEN | SXT | LVX | CIP | CTX | FEP | CAZ | CMZ | TZP | CSL | CZA | ATM | TGC | COL |
| J53 | *E.coli* | ≤0.125 | ≤0.125 | ≤0.125 | 4 | 1 | ≤0.125/2.4 | ≤0.125 | ≤0.125 | ≤0.25 | ≤0.25 | 0.5 | 2 | 4 | ≤1 | ≤1 | ≤1 | ≤0.125 | ≤0.125 |
| K24-J | *K. pneumoniae* | 32 | 8 | 16 | 2 | 0.5 | 0.25/4.7 | 0.25 | 0.25 | ＞32 | 32 | ＞32 | 64 | ＞256 | ＞128 | 16 | 8 | ≤0.125 | ≤0.125 |
| K32-J | *K. pneumoniae* | 8 | 8 | 16 | 1 | 0.25 | ≤0.125/2.4 | ≤0.125 | ≤0.125 | ＞32 | ＞32 | ＞32 | ＞64 | ＞256 | ＞128 | 32 | ≤1 | ≤0.125 | ≤0.125 |
| K158-J | *K. pneumoniae* | 16 | 16 | 32 | 1 | 0.5 | ≤0.125/2.4 | ≤0.125 | ≤0.125 | ＞32 | ＞32 | ＞32 | 64 | ＞256 | ＞128 | 16 | ≤1 | ≤0.125 | ≤0.125 |
| K27-J | *K. pneumoniae* | 8 | 16 | 16 | 1 | 0.25 | ≤0.125/2.4 | ≤0.125 | ≤0.125 | ＞32 | ＞32 | ＞32 | ＞64 | ＞256 | ＞128 | 64 | ≤1 | ≤0.125 | ≤0.125 |
| K176-J | *K. pneumoniae* | 8 | 8 | 32 | 1 | 0.5 | ≤0.125/2.4 | ≤0.125 | ≤0.125 | >32 | >32 | >32 | >64 | >256 | >128 | 16 | ≤1 | 0.25 | 0.5 |
| K178-J | *K. pneumoniae* | 32 | 16 | 32 | 1 | 0.25 | ≤0.125/2.4 | ≤0.125 | ≤0.125 | >32 | >32 | >32 | >64 | >256 | >128 | 16 | ≤1 | ≤0.125 | 0.25 |
| K182-J | *K. pneumoniae* | 64 | 32 | 32 | 2 | 0.25 | 0.25/4.7 | ≤0.125 | ≤0.125 | >32 | >32 | >32 | >64 | >256 | >128 | 64 | ≤1 | ≤0.125 | ≤0.125 |
| K183-J | *K. pneumoniae* | 16 | 8 | 32 | 0.5 | ≤0.125 | ≤0.125/2.4 | ≤0.125 | ≤0.125 | >32 | >32 | >32 | >64 | >256 | >128 | 16 | ≤1 | 0.25 | 0.25 |
| K184-J | *K. pneumoniae* | 8 | 32 | 16 | 2 | 0.5 | ≤0.125/2.4 | ≤0.125 | ≤0.125 | >32 | >32 | >32 | >64 | >256 | >128 | 64 | ≤1 | ≤0.125 | 0.25 |
| K161-J | *K. pneumoniae* | 16 | 32 | 8 | 2 | 0.5 | ≤0.125/2.4 | 0.25 | 0.25 | >32 | >32 | >32 | >64 | >256 | >128 | 32 | ≤1 | ≤0.125 | 0.25 |
| K185-J | *K. pneumoniae* | 32 | 16 | 16 | 1 | 0.5 | 0.5/9.5 | ≤0.125 | ≤0.125 | >32 | >32 | >32 | >64 | >256 | >128 | 32 | ≤1 | ≤0.125 | ≤0.125 |
| K187-J | *K. pneumoniae* | 32 | 32 | 8 | 1 | 0.5 | 0.25/4.7 | ≤0.125 | ≤0.125 | >32 | >32 | >32 | >64 | >256 | >128 | 64 | ≤1 | ≤0.125 | ≤0.125 |
| K45-J | *K. pneumoniae* | 16 | 8 | 32 | 4 | 0.5 | ≤0.125/2.4 | ≤0.125 | ≤0.125 | ＞32 | ＞32 | ＞32 | ＞64 | ＞256 | ＞128 | 32 | ≤1 | ≤0.125 | 0.25 |
| K96-J | *K. pneumoniae* | 64 | 16 | 32 | ≤0.25 | ≤0.125 | ≤0.125/2.4 | ≤0.125 | ≤0.125 | ＞32 | ＞32 | ＞32 | ＞64 | ＞256 | ＞128 | 32 | ≤1 | ≤0.125 | ≤0.125 |
| K702-J | *K. pneumoniae* | 16 | 8 | 16 | 1 | 0.25 | ≤0.125/2.4 | ≤0.125 | ≤0.125 | ＞32 | 16 | ＞32 | 64 | 256 | ＞128 | 64 | ≤1 | ≤0.125 | ≤0.125 |
| K725-J | *K. pneumoniae* | 32 | 8 | 16 | 1 | 0.25 | ≤0.125/2.4 | ≤0.125 | ≤0.125 | >32 | >32 | >32 | >64 | >256 | >128 | 16 | ≤1 | ≤0.125 | 0.25 |
| Z214-J | *E. coli* | 4 | 8 | 16 | 2 | 0.5 | ≤0.125/2.4 | ≤0.125 | ≤0.125 | >32 | >32 | >32 | >64 | >256 | >128 | 32 | ≤1 | ≤0.125 | ≤0.125 |
| Z244-J | *E. coli* | 16 | 8 | 32 | 2 | 0.5 | ≤0.125/2.4 | ≤0.125 | ≤0.125 | ＞32 | 32 | ＞32 | 64 | ＞256 | ＞128 | 16 | ≤1 | ≤0.125 | ≤0.125 |
| CR33-J | *K. aerogenes* | 32 | 64 | 64 | 2 | 0.25 | ≤0.125/2.4 | ≤0.125 | ≤0.125 | ＞32 | ＞32 | ＞32 | ＞64 | ＞256 | ＞128 | 16 | ≤1 | ≤0.125 | ≤0.125 |
| CR94-J | *K. aerogenes* | 16 | 8 | 8 | 1 | 0.5 | ≤0.125/2.4 | ≤0.125 | ≤0.125 | >32 | >32 | >32 | >64 | >256 | >128 | 16 | ≤1 | ≤0.125 | 0.25 |
| CR39-J | *K. aerogenes* | 8 | 32 | 32 | 2 | 0.5 | ≤0.125/2.4 | ≤0.125 | ≤0.125 | >32 | >32 | >32 | >64 | >256 | >128 | 64 | ≤1 | ≤0.125 | 0.25 |
| CR50-J | *K. aerogenes* | 16 | 8 | 32 | 2 | 0.25 | ≤0.125/2.4 | ≤0.125 | ≤0.125 | ＞32 | ＞32 | ＞32 | ＞64 | ＞256 | ＞128 | 16 | ≤1 | ≤0.125 | ≤0.125 |

Abbreviations: ETP, ertapenem; IPM, imipenem; MEM, meropenem; AMK, amikacin; GEN, gentamicin; SXT, sulfamethoxazole-trimethoprim; CIP, ciprofloxacin; LVX, levofloxacin; CTX, cefotaxime; FEP, cefepime; CAZ, ceftazidime; CMZ, cefmetazole; TZP, piperacillin-tazobactam; CSL, cefoperazone-sulbactam; CZA, ceftazidime-avibactam; ATM, aztreonam; TGC, tigecycline; COL, colistin.
